# Hemagglutinin stalk-based monoclonal antibody elicits broadly reactivity against group 1 influenza A virus

**DOI:** 10.1186/s12985-020-01458-z

**Published:** 2020-12-07

**Authors:** Jingjin Huang, Nan Huang, Menglu Fan, Lingcai Zhao, Yan Luo, Pingyun Ding, Miao Tian, Qingzheng Liu, Yanna Guo, Jinhua Zhao, Yiqing Zheng, Haitao Zhang, Jihui Ping

**Affiliations:** 1 MOE International Joint Collaborative Research Laboratory for Animal Health and Food Safety & Jiangsu Engineering Laboratory of Animal Immunology, College of Veterinary Medicine, Nanjing Agricultural University, Nanjing, 210095 China; 2 Biotechnology Research laboratory, Jiangsu Lihua Animal Husbandry co. LTD, Changzhou, 213168 China

**Keywords:** Influenza virus, HA2 stalk region antibody, Broad-spectrum, Epitope

## Abstract

**Background:**

Influenza virus remains a continuous and severe threat to public health worldwide, and its prevention and treatment have always been a major international issue. Because of its ability to evade immune surveillance through rapid antigenic drift and antigenic shift, broad-spectrum vaccines seem increasingly important.

**Methods:**

A mAb named 3C12 from an immortalized hybrid cell was generated via immunizing mice with HA2 protein from A/chicken/Anhui/BRI99/2016 (AH/BRI99/16, H9N2) generated by prokaryotic expression. Then, its broad-spectrum activity was analyzed by WB and IFA. Next, the minimal linear epitope was identified via analyzing the reaction of a series of HA truncations with 3C12. Finally, the protective effects of 3C12 were evaluated in vitro and in vivo infection experiments.

**Results:**

The mAb could react with the viruses of subtypes H1, H2, H5, H8, H9, H12, H13, H16, and HA protein of H18 in group 1, but failed to react with viruses in group 2. The minimal linear epitope targeted by the mAb was ^433^NAELLVL^439^ in full length of HA and localized in the C-helix region of HA2 (residue 95-101, HA2 numbering). What’s more, the mAb 3C12 inhibited H1, H2, H5, H8, H9, H12, H13 and H16 virus-replication in vitro and also has shown effectiveness in preventing and treating disease in mice challenged with lethal dose of AH/BRI99/16 (H9N2) virus in vivo. These results suggested that the broadly reactive anti-HA stem mAb 3C12 exhibited prophylactic and therapeutic efficacy.

**Conclusions:**

Here, we have demonstrated that the linear epitope identified in this study could be a novel target for developing broad-spectrum influenza diagnostics or vaccine design, and the HA2-based monoclonal antibody is indeed a promising strategy for broad-spectrum protection against seasonal and pandemic influenza viruses.

## Background

Influenza viruses cause millions of cases of severe illness, thousands of deaths, and considerable economic losses each year [1]. According to the collecting data from World Health Organization (WHO), influenza A viruses (IAVs) annually cause about 3 to 5 million cases of severe illness and approximately 290,000 to 650,000 respiratory deaths worldwide [2]. Importantly, some avian influenza viruses have the potential to acquire mammalian transmissibility by reassortment, such as H5N1 [2]. And some vaccines against H5N1 viruses, including inactivated vaccines and live virus vectored vaccines were produced to protect the avian and human [3, 4]. IAVs possess eight segmented, negative-sense viral RNAs (vRNAs) as its genome. Two of these vRNAs encode hemagglutinin (HA) and neuraminidase (NA), which are major viral antigenic proteins on the virus particle [5]. HA is a trimer of HA1 and HA2 that is produced by cleavage of the precursor HA0 [6] and belongs to type I glycoprotein, which is the most abundant transmembrane protein on the surface of influenza viral particles [7]. On the basis of the two major surface antigenic proteins HA and NA, IAVs can be currently subtyped into 18 HA and 11 NA serotypes, respectively [8, 9]. The HA1 subunit of HA mediates attachment of the virus to target cells through interactions with sialic acid receptors. After the virus was devoured, the low pH triggers conformational changes in HA2, leading to fusion of the viral and endosomal membranes, releasing the viral genome into the cytoplasm [1]. So far, some HA inhibitors that block the fusion of HA with endosome have already been generated, seen in the following list: CL-61917, CL-385319, and CL-62554 [10]; BMY-27709 [11]; RO5464466 and RO5487624 [12]; FA-583 and FA-617 [13] which target group 1 HAs; whereas TBHQ [14, 15], S19 and C22 [16] are fusion inhibitors to group 2 HAs. However, excessive use leads to resistant strains [17–19] that often show surprisingly little attenuation from the escape mutations. Considering the high variability and rapid microevolution of the influenza virus, vaccination remains the most effective countermeasure against influenza outbreak. Notably, current commercial vaccines of IAVs are still strain-specific and show only limited protective efficacy against the emerging strains with antigenic drift or shift [20, 21]. Therefore, a broad-spectrum vaccine against all the 18 HA subtypes is highly required for protection against epidemics or pandemics of IAVs in both human and animals.

The HA2 subunit makes up the major part of the HA stalk region and is highly conserved within subtypes [22, 23]. Subsequently, some monoclonal antibodies owning heterosubtypic activity have already been generated. According to the reactivity, the HA2 target mAbs were divided into three types. The first type contains monoclonal antibodies that react with group 1; mAbs CR6261 [24, 25], F10 [26], 3.1 [27], FE43, FC41 [28], A06 [29] and PN-SIA49 [30] belong to the first type. The second type contains monoclonal antibodies CR8020 [1], CR8043 [31], 9H10 [32], 042-100,809-2F04 [33] and SD36 [34] that react with group 2. The final type contains monoclonal antibodies reacted with both group 1 and group 2 including CR9114 [35]; FI6v3 [36], 39.29 and CT149 [37], PNSIA28 [38], VIS410 [39], MEDI8852 [40], 27F3 [41], S9-1-10/5-1 [5] and SD38 [34]. Furthermore, CR9114 [35] and 27F3 [41] could also react with the HA stem region of influenza B virus.

Here, we generated a HA2-based monoclonal antibody named 3C12. The region recognized by 3C12 was localized to a conserved portion of the HA stem domain. 3C12 can react with a large amount of subtypes of group 1 viruses including H1, H2, H5, H8, H9, H12, H13, H16 and also HA protein of H18. Next, we mapped the epitope of 3C12 ^95^NAELLVL^101^ (HA2 numbering) located in the C-helix region following the loop of HA2. 3C12 could inhibit infection of MDCK cells from H1, H2, H5, H8, H9, H12, H13 and H16 viruses in vitro. Additionally, mAb 3C12 showed prophylactic and therapeutic efficacy in mouse models challenged with a lethal dose of AH/BRI99/16 (H9N2). Therefore, we demonstrated that the linear epitope identified in this study could be a novel target for developing broad-spectrum influenza diagnostics or vaccine candidates, and that the HA2-based monoclonal antibody is a promising strategy for broad-spectrum protection against seasonal and pandemic influenza viruses.

## Methods

### Viruses and plasmids

The wild type or recombinant influenza viruses used in this study were as follows: A/duck/Alberta/35/76 (Alberta/35/76, H1N1), A/gull/Maryland/19/1977 (Maryland/19/77, H2N9), A/duck/Ukraine/1/1963 (Ukraine/1/63, H3N8), A/duck/Czechoslovakia/1956 (Czechoslovakia/56, H4N6), A/turkey/Wisconsin/1/1968 (Wisconsin/1/68, H5N9), A/turkey/Massachusetts/3740/1965 (Massachusetts/3740/65, H6N2), A/turkey/Oregon/1971 (Oregon/71, H7N3), A/turkey/Ontario/6118/1968 (Ontario/6118/68, H8N4), A/chicken/Anhui/BRI99/2016 (AH/BRI99/16, H9N2), A/chicken/Germany/N/1949 (Germany/N/49, H10N7), A/duck/England/1/1956 (England/1/56, H11N6), A/duck/Alberta/60/1976 (Alberta/60/76, H12N5), A/gull/Maryland/704/1977 (Maryland/704/77, H13N6), A/mallard/Astrakhan/263/1982 (Astrakhan/263/82, H14N5), A/shearwater/West Australia/2576/79 (Australia/2576/79, H15N9), A/black-headed gull/Sweden/5/99 (Sweden/5/99, H16N3), A/little yellow-shouldered bat/Guatemala/164/2009 (Guatemala/164/09, H17N10), a/flat-faced bat/ Peru/033/2010 (Peru/033/2010, H18N11), A/PuertoRico/8/1934 (PR8, H1N1), r-PR8 + California/04/2009 (HA + NA) (r-PR8-Cal/04, H1N1), A/WSN/1933 (WSN, H1N1), A/turkey/Wisconsin/1/1966 (Wisc/66, H9N2), A/chicken/Shandong/6/1996 (SD/6/96, H9N2), A/chicken/Guangxi/55/2005 (GX/55/05, H9N2), A/chicken/Guangdong/1/2010 (GD/1/10, H9N2), A/chicken/Jiaxing/36/2013 (JX/36/13, H9N2), A/chicken/Shandong/32/2015 (SD/32/15, H9N2), A/chicken/Anhui/LH66/2017 (AH/66/17, H9N2), A/chicken/Changzhou/2/2019 (CZ/2/19, H9N2), B/Jiangsu/103/2015 (B/JS/103/15, Flu B). They were generated by reverse genetics or isolated from infected poultries and then inoculated into 10-day-old specific-pathogen-free (SPF) chicken embryos for virus propagation. The HA segment of H17 and H18 were synthesized (General Bio-systems, Anhui) and cloned into pCAGGS protein expressing vector.

### Cells

Two hundred ninety-three T human embryonic kidney cells obtained from American Type Culture Collection (ATCC) were maintained in DMEM supplemented with 10% fetal bovine serum. Madin-Darby Canine Kidney (MDCK) cells (obtained from ATCC) were maintained in DMEM containing 10% new born calf serum.

### Virus rescue

Virus rescues were performed as previously described by the twelve-plasmid reverse genetics system in a A/Puerto Rico/8/1934 (PR8) backbone [42]. Briefly, 1 μg of each protein expression plasmid (pCAGGS-WSN-PB2, pCAGGS-WSN-PB1, pCAGGS-WSN-PA and pCAGGS-WSN-NP) and 0.2 μg of each viral RNA transcription plasmid (pHH21-PR8-PB2, pHH21-PR8-PB1, pHH21-PR8-PA, pHH21-PR8-NP, pHH21-PR8-NA, pHH21-PR8-M, pHH21-PR8-NS and pHH21-H1-HA to pHH21-H16-HA plasmid) were combined with 12 μl Lipofectamine 2000 (2 μl per μg DNA, Invitrogen), and the mixture was incubated at room temperature for 30 mins and then transferred to 80% confluent monolayers of 293 T cells in 35 mm dish. After 6 h, the mixture was removed from the 293 T cells and replaced with Opti-MEM (Gibco-BRL). Forty-eight hours after transfection, the culture medium was collected and inoculated into 10-day-old SPF chicken embryos for virus propagation.

### Expression of recombinant proteins

The HA2 gene of AH/BRI99/16 (H9N2) was amplified by RT-PCR using the gene specific primers. Then the fragment was amplified with the specific program as follows: 5 min at 95 °C for predenaturation; 35 cycles of 30 s at 95 °C for denaturation, 30 s at 54 °C for annealing, 1 min at 72 °C for elongation; and finally, 10 min at 72 °C for overall elongation. The obtained HA2 fragment was subcloned into the prokaryotic expression vector pET-28a (Takara, Japan). The recombinant plasmid was verified by Sanger sequencing and then transformed into *E. coli* BL21 cells for the expression of HA2 protein. The expression of H9-HA2 protein including His tag was induced with 1 mM isopropyl β-D-1- thiogalactopyranoside (IPTG), and the protein was purified using a Ni-NTA agarose (Thermo, USA) according to the manufacturer’s instructions. The purified fusion protein was identified with SDS-PAGE and Western blot.

### Preparation of anti-HA2 protein monoclonal antibodies

The mAbs were prepared as described in a previous study [43]. The recombinant H9-HA2 protein was used as the immunogen for the development of mAbs in this study. Briefly, six-week-old female BALB/c mice were injected subcutaneously with the mixture of 50 μg of the purified recombinant HA2 protein and an equal volume of complete Freund’s adjuvant (Sigma-Aldrich, USA). And the mice were intraperitoneally immunized with the mixture of the purified recombinant HA2 protein and an equal volume of incomplete Freund’s adjuvant at 14 dpi and 28 dpi. Three days after the last immunization, the mice were boosted with 100 μg of the HA2 protein, the spleen cells of the best responder animals were harvested and fused with SP2/0 myeloma cells using polyethylene glycol 2000 (Sigma-Aldrich, USA) according to the manufacture’s instruction. The fused cells were selected in hypoxanthine, aminopterin and thymidine (HAT) medium. The positive clones were screened by ELISA using the recombinant HA2 protein and were subcloned 4 times by limiting dilution. The mAbs against H9-HA2 were collected and purified from the mouse ascites, which injected with the positive hybridomas.

### Construction of the HA amino acid sequence dendrogram

The HA genes of all H1-H18 subtypes used in this study were aligned by MegAlign (Clustal W Method) and beautified by iTOL website.

### Western blot

MDCK cells were infected with the H1-H16 subtype corresponding recombinant viruses or 293 T cells were transfected with pCAGGS-H17HA or pCAGGS-H18HA plasmid. Cells were collected and lysed with NP-40 lysis buffer (Beyotime, Shanghai) after 24 h’ infection or transfection. Cell lysates were mixed with 4 × loading buffer (Solarbio, Beijing) and denatured at 100 °C for 15 min, then were separated using SDS-PAGE with a 10% polyacrylamide gel [44] and transferred to NC membranes (GE Healthcare, Amersham). Next, membranes were blocked with 5% skimmed milk in PBS at 37 °C f for 1 h, then washed six times (5 min per time) with PBST and incubated with primary antibody 3C12 (1:800) at 4 °C overnight. Then, after six times washing, the membrane was incubated with HRP-conjugated goat anti-mouse antibody (1:10000; KPL, Gaithersburg, MD) as secondary antibody at 37 °C for 1 h. Membranes were then washed and the target protein bands were detected with enhanced chemiluminescence (ECL) (Vazyme, Nanjing) and the signals were recorded using Image Lab Software (Bio-Rad). GADPH served as a loading control.

### Indirect immunofluorescence assay

For the indirect immunofluorescence assay, 80% confluent MDCK cells in 12-well plate were inoculated with the viruses mentioned above at MOI = 0.01 at 37 °C for 1 h, then the virus inoculum was removed and replaced by cell maintenance medium with 1 μg/ml TPCK-trypsin and were incubated at 5% CO_2_, 37 °C for 24 h. In addition, the 70% confluent 293 T cells in 12-well plate were transfected with 500 ng pCAGGS-H17 HA or pCAGGS-H18 HA plasmids for 24 h. Then the infected MDCK cells or transfected 293 T cells were washed with PBS, fixed with 4% paraformaldehyde for 20 mins at room temperature, permeabilized with 0.2% Triton X-100 (Sigma-Aldrich, USA) in PBS for 15 min, and then washed with PBS for three times. 5% skimmed milk in PBS was then added to the desired wells for 30 mins at 37 °C. Then, the cells were washed and stained with primary antibody 3C12 (1:800) at 4 °C overnight. After six times washing, the cells were incubated with fluorescein isocyanate (FITC)-conjugated goat anti-mouse IgG (1:500; KPL, Gaithersburg, MD, USA) as secondary antibody at 37 °C for 1 h. Finally, images were acquired using fluorescent microscope (Nikon, Tokyo, Japan).

### Mapping of HA2 protein linear epitope

To identify the precise epitopes of the HA2 protein targeted by 3C12, the HA gene of AH/BRI99/16 was truncated step by step. These truncated fragments of AH/BRI99/16 HA were amplified and cloned into the pCAGGS protein expressing vector. To verify whether the minimal amino acids were the shortest epitope, the gene of short peptides were amplified and cloned into the pGEX-4T-1 vector containing the GST tag and expressed in *E. coli* BL21 (DE3). Then, the truncated HA fragments and short peptides were transfected into 293 T cells, the cells were lysated or fixed at 24 h post-transfection and then incubated with mAb 3C12 for WB or IFA analysis.

### Alignment of HA2 protein epitope sequences

The alignment of amino acid sequences of HA2 target epitope was performed by MEGA6 program.

### Micro-neutralization assay of mAb 3C12 in vitro

To test the neutralization activity of 3C12, the mAb was 2-fold serial diluted in 96-well plates. One hundred TCID_50_ of corresponding viruses were mixed with diluted 3C12, then the mixture was incubated at 37 °C for 1 h. After that, the mixture was inoculated into 95% confluent MDCK cells in 96-well plates. After 1 h incubation, the infected MDCK cells were washed with PBS and then cultured in DMEM with 1 μg/ml TPCK-trypsin at 37 °C for 3 days. After that the neutralizing titers were measured by hemagglutination assay (HA). The results were evaluated by the value of IC_50._

### Evaluation of mAb 3C12 for its prophylactic and therapeutic protective activities in mice model

To identify the prophylactic and therapeutic efficacy in mice, six to eight-week-old female BALB/c mice were used in this study. Mice were weighed the body weight on the day or 1 day before virus challenge and monitored daily until day 14 for weight loss and survival (mice with body weight loss of ≥25% were euthanized).

For the prophylactic efficacy study, five groups mice (five mice of each group) were intravenously injected with mAb 3C12 at a dose of 1 mg/kg, 5 mg/kg, 10 mg/kg, 20 mg/kg in 50 μl volume or PBS as a control. Three hours after mAb administration, all the mice were intranasally inoculated with 10 MLD_50_ AH/BRI99/16 (H9N2) in 50 μl volume. For the therapeutic efficacy study, seven groups mice (five mice per group) were intranasally inoculated with 10 MLD_50_ AH/BRI99/16 (H9N2) virus, then the mice received 15 mg/kg mAb 3C12 treatment intravenously (i.v.) on day 0, 1, 2, 3, 4, 5 days post-infection, or PBS treatment as control.

### Ethical statement

The mice were handled humanely according to the rules described by the Animal Ethics Procedures and Guidelines of the People’s Republic of China and Institutional Animal Care and Use Committee of Nanjing Agricultural University [SYXK(Su)2017-0007].

## Results

### Identification of monoclonal antibody binding to influenza A viruses of group 1

**Fig. 1:**
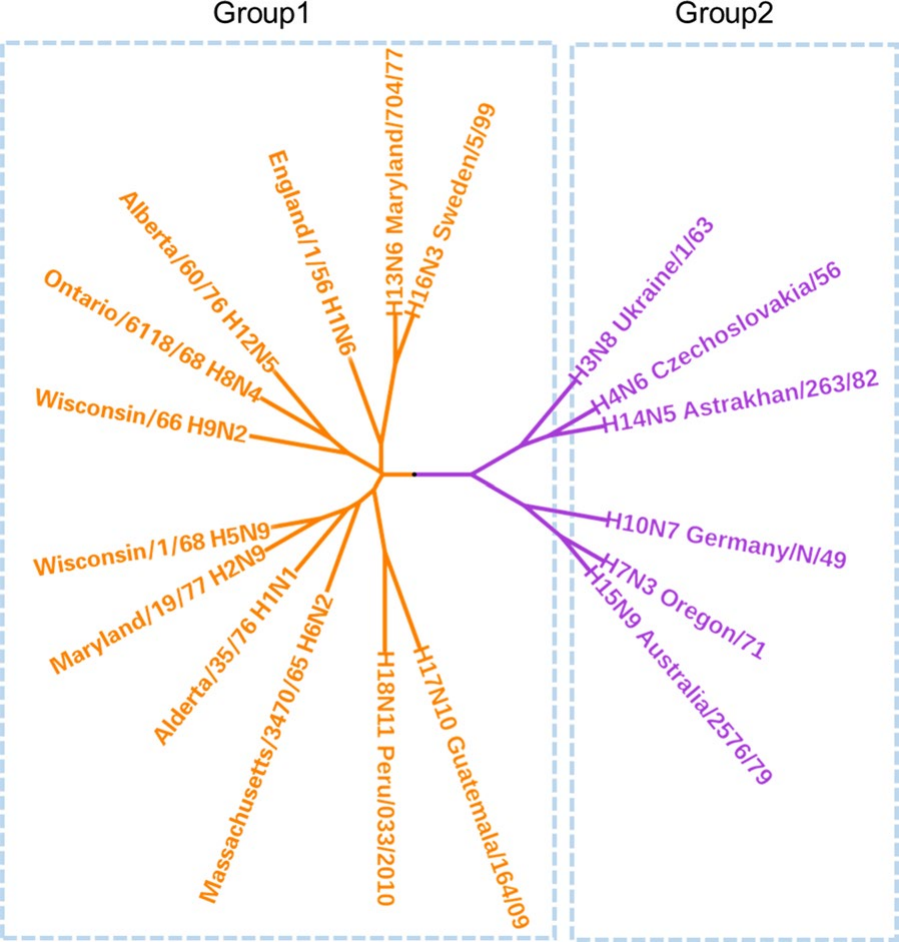
The phylogenetic tree of the H1-H18 HA sequences tested in this study. The phylogenetic tree was made by the neighbor-joining method. Group 1 subtypes were shown in orange and group 2 subtypes in purple. They stood for the short name of the strains

**Fig. 2:**
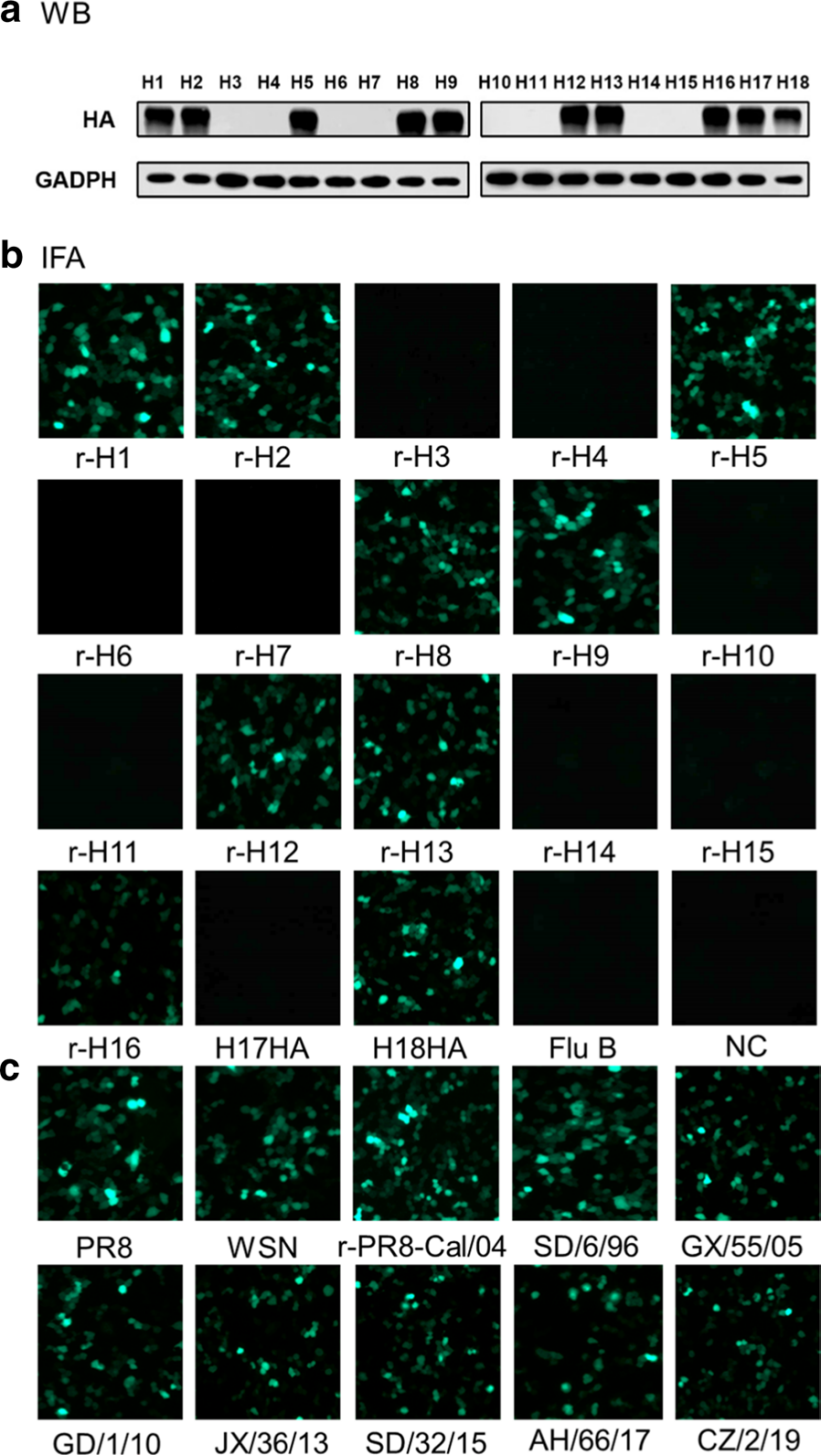
The broad-spectrum neutralization of influenza viruses against mAb 3C12. **a** Western Blot (WB) analysis. The lysates of influenza virus H1-H16-infected MDCK cells and H17 and H18 HA protein transfected 293 T cells were incubated with the mAb 3C12. **b** Indirect immunofluorescence assays (IFA). MDCK cells were infected with H1-H16 and flu B for no more than 24 h, 293 T cells were transfected with pCAGGS-H17 HA and H18 HA for 24 h, and then the cells were fixed and incubated with mAb 3C12. **c** IFA. MDCK cells were infected with other strains of subtype H1 and H9. NC served as negative control

In this study, we generated several cross-reactive mouse monoclonal antibodies. One of which, named 3C12, could react with several different influenza A viruses. To identify exactly which HA subtypes could be recognized by mAb 3C12, influenza A viruses of subtypes H1 through H16 and HA proteins for H17 and H18 (because of failure of generation H17 and H18 recombinant viruses) were employed for reactivity testing. The different subtypes of influenza A viruses tested in this study were shown in a phylogenetic tree which separated into group 1 and group 2 (Fig. 1). The binding activity of the mAb was examined by western blot (WB) and indirect immunofluorescence assay (IFA). Both results showed that the mAb could react with many influenza subtypes. According to WB data (Fig. 2a), we found that mAb 3C12 was able to react with H1, H2, H5, H8, H9, H12, H13, H16 viruses and the H17, H18 HA proteins. The IFA data largely confirmed the WB results, except for the H17 HA protein (Fig. 2b). These data suggested that 3C12 could react with a majority of viruses of group 1, however, it did not react with any viruses of group 2. The selected mAb 3C12 was also tested against a panel of human H1N1 viruses spanning more than 80 years, and H9N2 representatives strains of different antigenic groups. Specifically, PR8, WSN, and r-PR8-Cal/04 were chosen to represent H1N1 subtypes; SD/6/96, GX/55/05, GD/1/10, JX/36/13, SD/32/15, AH/66/17 and CZ/2/19 were chosen to represent H9N2 subtypes. Interestingly, the mAb 3C12 could react with all of these viruses (Fig. 2c). Detailed information about the viruses used in this study were shown in Table 1. Summarizing the results above, the mAb 3C12 could not only react with numerous viruses of group 1, but also with various different isolates of H1 and H9 subtypes in different years with different antigenicity, including both North-American and Eurasian lineages of H9N2 strains.

**Table 1:** Names of viruses used in this article

Subtype	Full name	Short name
H1	r-PR8-A/duck/Alberta/35/76	Alberta/35/76
	A/Puerto Rico/8/1934	PR8
	A/WSN/1933	WSN
	r-PR8-California/04/2009	r-PR8-Cal/04
H2	r-PR8-A/gull/Maryland/19/1977	Maryland/19/77
H3	r-PR8-A/duck/Ukraine/1/1963	Ukraine/1/63
H4	r-PR8-A/duck/Czechoslovakia/1956	Czechoslovakia/56
H5	r-PR8-A/turkey/Wisconsin/1/1968	Wisconsin/1/68
H6	r-PR8-A/turkey/Massachusetts/3740/1965	Massachusetts/3740/65
H7	r-PR8-A/turkey/Oregon/1971	Oregon/71
H8	r-PR8-A/turkey/Ontario/6118/1968	Ontario/6118/68
H9	r-PR8-A/chicken/Anhui/BRI99/2016	AH/BRI99/16
	r-PR8-A/chicken/Shandong/6/96	SD/6/96
	r-PR8-A/chicken/Guangxi/55/2005	GX/55/05
	r-PR8-A/chicken/Guangdong/1/2010	GD/1/10
	r-PR8-A/chicken/Jiangxi/36/2013	JX/36/13
	r-PR8-A/chicken/Shandong/32/2015	SD/32/15
	r-PR8-A/chicken/Anhui/LH66/2017	AH/66/17
	A/chicken/Changzhou/2019	CZ/2/19
H10	r-PR8-A/chicken/Germany/N/1949	Germany/N/49
H11	r-PR8-A/duck/England/1/1956	England/1/56
H12	r-PR8-A/duck/Alberta/60/1976	Alberta/60/76
H13	r-PR8-A/gull/Maryland/704/1977	Maryland/704/77
H14	r-PR8-A/mallard/Astrakhan/263/1982	Astrakhan/263/82
H15	r-PR8-A/shearwater/West Australia/2576/79	Australia/2576/79
H16	r-PR8-A/black-headed gull/Sweden/5/99	Sweden/5/99
H17	A/little yellow-shouldered bat/Guatemala/164/2009	Guatemala/164/09
H18	A/flat-faced bat/Peru/033/2010	Peru/033/10

### Mapping of the potential cross-protective epitope on HA protein

**Fig. 3:**
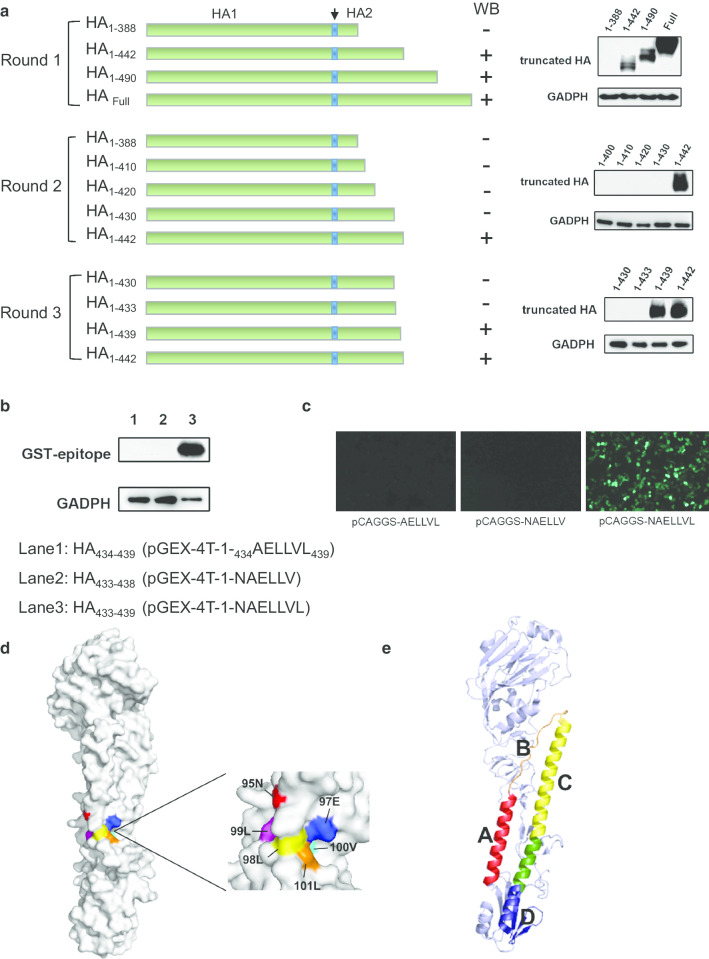
Analysis of the HA2 protein linear epitopes using WB and IFA. **a** Schematic representation of the HA truncated fragments used for epitope mapping (left). The corresponding results of WB were shown in right and the reactive fragments were containing ^433^NAELLVL^439^ (or ^95^NAELLVL^101^, HA2 numbering) sequence in full length of HA. **b** The minimal epitope mapping of 3C12 using WB. The 433-439aa sequences was cloned into pGEX-4 T-1 vector and expressed in *E. coli* BL21 then subjected to incubation with mAb 3C12. **c** Reaction of the mAb with the minimal epitope were evaluated by IFA. The three epitopes were cloned into pCAGGS vector and transfected into 293 T cells, respectively. **d** The location of the epitope recognized by 3C12 in the HA molecule was determined by PyMOL using the PDB 1jsd (H9N2 HA). The corresponding positions were colored and pointed out with lines. Since the Alanine residue of the position 96 (HA2) did not exist on the surface, we could not map it. All the residues were represented by HA2 numbering. **e** The structure of HA molecule showed by cartoon using the PDB 1jsd. HA with helicx A (red), C (yellow), and D (blue) and B loop (orange) in one monomer colored distinctly

To identify the target epitope of mAb 3C12, a series of truncated HA fragments of AH/BRI99/16 (H9N2) were constructed and the reaction activity was evaluated by WB. Based on the first round truncation, we found the mAb recognized the fragment HA_388-490_ (HA2_50-152_) well. Following another round truncation, the mAb was confirmed to recognize the fragment HA_430-442_ (HA2_92-104_). After that, the truncated fragment was then shortened to HA2_95-101_, and the WB data showed the ^95^NAELLVL^101^ (HA2 numbering) could still be bound by 3C12, see Fig. 3a. Next, we were wondering to know if this polypeptide was the shortest target epitope, so the fragments 432-439 aa (HA2_94-101_), 433-439 aa (HA2_95-101_), 433-438 aa (HA2_95-100_) of AH/BRI99/16 HA were cloned into the pGEX-4T-1 vector and expressed in E. coli BL21 (DE3), then the reactivity was verified by WB (Fig. 3b). Furthermore, the fragment HA2_95-101_ aa was also cloned into pCAGGS expressing vector and transfected 293 T cells to identify the fluorescent through IFA (Fig. 3c). Both WB and IFA results demonstrated mAb 3C12 bound to the ^95^NAELLVL^101^ (HA2 numbering) epitope. The localization of the epitope recognized by the mAb was highlighted in the three-dimensional (3D) structure of HA protein (Fig. 3d). Since the 94A residue is not on the HA surface, it could not be represented. The epitope was displayed in cartoon format to identify the specific region recognized by the mAb. The epitopes of many previously studies showed that HA2-based mAbs were mainly located in fusion peptide and helix A region. Encouragingly, we found that the epitope was located in the relatively conserved C-helix region of HA2 (Fig. 3e).

### Alignment of HA protein epitope sequences and its structure

**Fig. 4:**
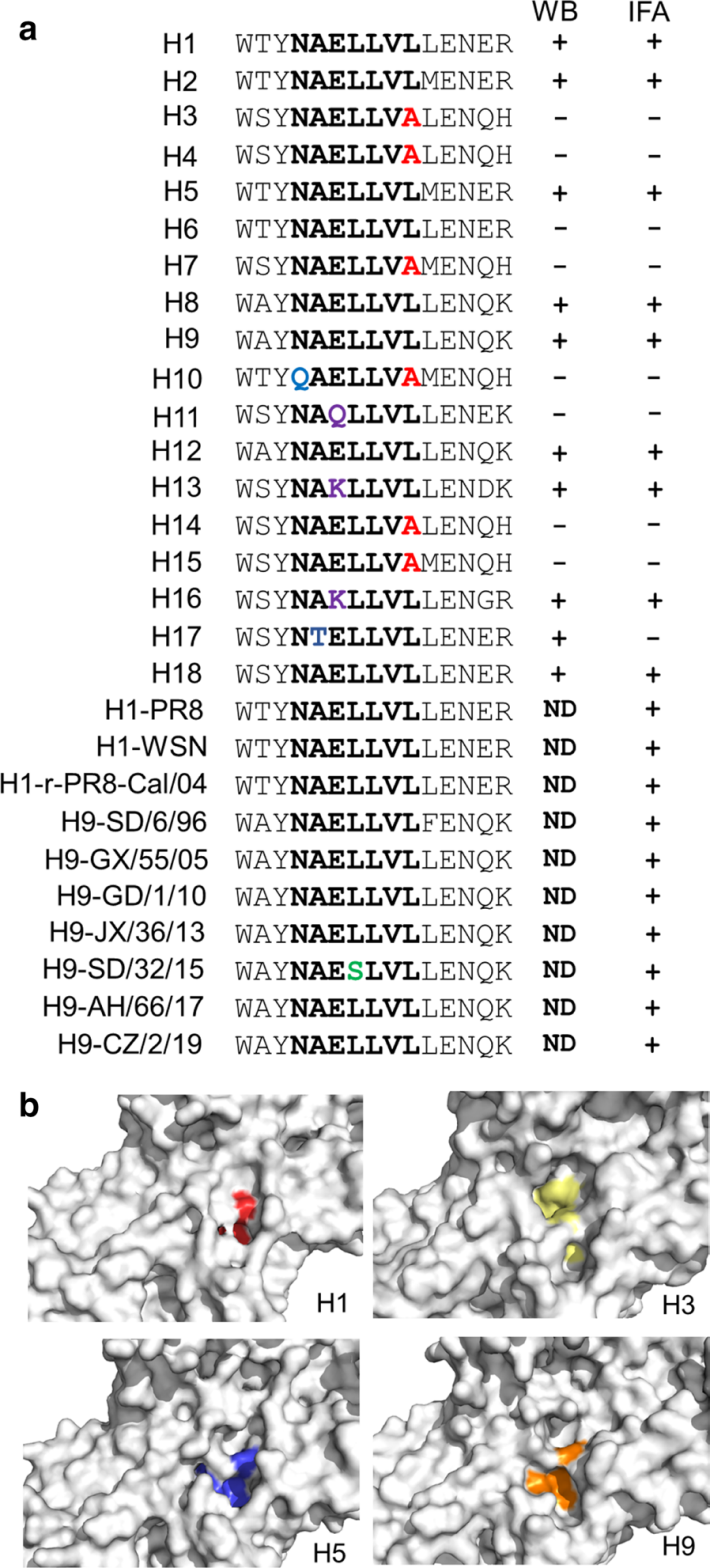
Amino acid difference of the epitope of all the viruses used in the study. **a** The minimal epitope and its nearby amino acids were shown, with the minimal epitope in bold and mutations in colored. The reactivities of WB and IFA were shown on the right panel. “+” stood for reactivity, “-” stood for non-reactivity. ND stood for not done. **b** The location of the epitope recognized by 3C12 in the H1, H3, H5 and H9 HA protein 3D structures was generated by PyMOL. H1, H3, H5 and H9 were shown in red, yellow, blue and orange respectively. H1 PDB number: 2WRH; H3 PDB number: 2HMG; H5 PDB number: 2IBX

Influenza A viruses can be separated into two phylogenetic groups according to the sequence characteristic of HA2, known as group 1 and group 2. So, we next explored the reason why mAb 3C12 was able to recognize the subtypes in group 1, but not the subtypes of group 2. Firstly, we analyzed the reason for the HA2 liner sequence level. Through alignment of the HA2 gene sequences from H1-H18 subtypes, we found that the epitope ^95^NAELLVL^101^ (HA2 numbering) is quite conserved in the majority of subtypes of group 1. In contrast, the HA subtypes of group 2 are all ^95^NAELLVA^101^ (HA2 numbering) except H10, which deviates from the group at N95Q (HA2 numbering) (Fig. 4a). Given this, we speculated that L101A (HA2 numbering) eliminated the binding of the mAb with mutant epitopes, limiting its reactivity across groups. In those group 1 subtypes without mAb 3C12 reactivity, including H6, H11 and H17, the single mutation of E97Q (HA2 numbering) or A96T (HA2 numbering) may alter the epitope recognition. It is surprisingly that the epitope of H6 HA was the same as the sequence of H9 HA, but the mAb 3C12 did not react with the H6 strain we selected in this study. Moreover, there may be other residues in HA2 protein that blocked its reactivity. Next, we investigated the protein trimeric structure differences among these subtypes. Interestingly, the C-helix region was partly located in the interior of HA trimer. And the structure of H1, H3, H5 and H9 subtypes were presented in Fig. 4b.

### Micro-neutralization assay of the 3C12 mAb in vitro

To evaluate the effect of preventing infection of 3C12 in vitro, we measured its neutralization ability with H1-H16 recombinant viruses in MDCK cells. According to the results shown in Table 2, it can be seen that mAb 3C12 has the strongest inhibition ability with a half maximal inhibitory concentration (IC_50_) value of 17.4 μg/ml against AH/BRI99/16 (H9N2) and Alberta/60/76 (H12) viruses. It has intermediate levels of inhibitory ability against H2, H13, H16 and two hererologous H9N2 viruses, and low inhibitory ability against H1, H5 and H8 viruses. Unfortunately, the 3C12 mAb didn’t show any neutralization ability against group 2 influenza A viruses (Table 2).

**Table 2:** Micro-neutralization activity of the mAb 3C12

Group	Subtype	Virus name	IC_50_ (μg/ml)
Group 1	H1	rPR8-A/duck/Alberta/35/76	52.1
	H2	rPR8-A/gull/Maryland/19/1977	34.7
	H5	rPR8-A/turkey/Wisconsin/1/1968	52.1
	H6	rPR8-A/turkey/Massachusetts/3740/1965	ND
	H8	rPR8-A/turkey/Ontario/6118/1968	52.1
	H9	rPR8-A/chicken/Anhui/BRI99/2016	17.4
		rPR8-A/chicken/Guangdong/1/2010	34.7
		rPR8-A/chicken/Shandong/32/2015	34.7
	H11	rPR8-A/duck/England/1/1956	ND
	H12	rPR8-A/duck/Alberta/60/1976	17.4
	H13	rPR8-A/gull/Maryland/704/1977	34.7
	H16	rPR8-A/black-headed gull/Sweden/5/1999	34.7
Group 2	H3	rPR8-A/duck/Ukraine/1/1963	ND
	H4	rPR8-A/duck/Czechoslovakia/1956	ND
	H7	rPR8-A/turkey/Oregon/1971	ND
	H10	rPR8-A/chicken/Germany/N/1949	ND
	H14	rPR8-A/mallard/Astrakhan/263/1982	ND
	H15	rPR8-A/shearwater/West Australia/2576/79	ND

*ND* not detection

### Prophylactic and therapeutic efficacy of 3C12 mAb in vivo

**Fig. 5:**
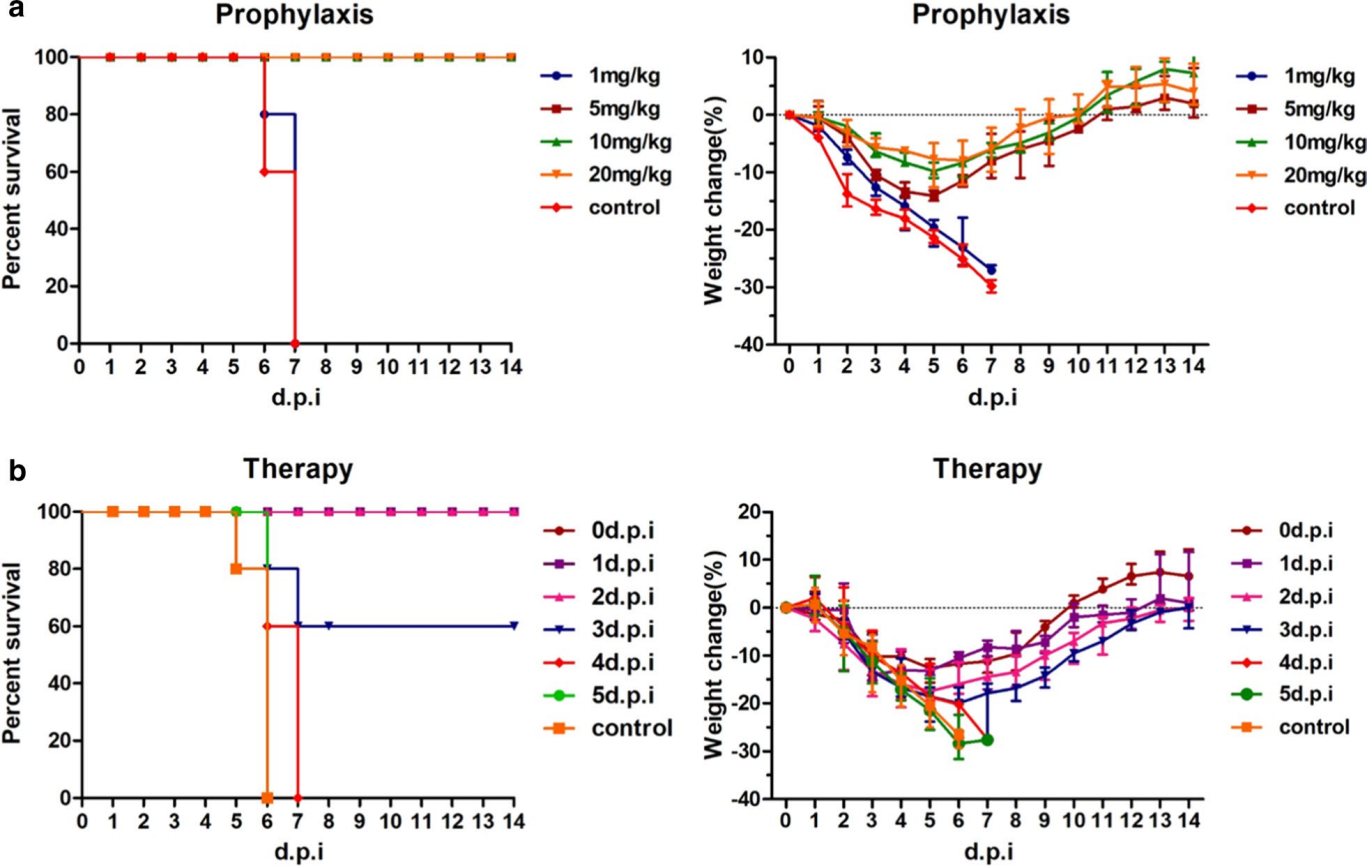
Prophylactic and therapeutic efficacy of 3C12 in mice. **a** Survival curves (left) and body weight loss (right) of BALB/c mice (five mice per experimental group) that received different concentrations 1 mg/kg, 5 mg/kg, 10 mg/kg, 20 mg/kg of 3C12 or PBS as a control intravenously (i.v.) 3 h before intranasal infection with 10-fold doses lethal to 50% of mice (MLD_50_) of AH/BRI99/16 virus. **b** Survival curves (left) and body weight loss (right) of mice (five per experimental condition) that received 15 mg/kg of 3C12 i.v. on day 0 (3 h before infection) or on day 1, 2, 3, 4, 5 post infection with 10 MLD_50_ AH/BRI99/16 virus. PBS as a control

To evaluate the prophylactic and therapeutic efficacy of mAb 3C12, the grouped mice were injected with 3C12 antibody and then challenged with lethal doses of recombinant virus or vice versa. In a prophylactic model, 5 mg/kg mAb 3C12 could fully protect mice from lethal infection (Fig. 5a), which consistent with the absence of any signs of respiratory distress in mice. However, the mice that received PBS rapidly lost weight, showed signs of respiratory distress, and succumbed to infection or were euthanized when mice had lost 25% of their body weight. While verifying the therapeutic efficiency, at a dose of 15 mg/kg mAb 3C12 was able to completely prevent mortality when administered on day 1 and 2 post-infection with 10 MLD_50_ AH/BRI99/16 (H9N2) challenge (Fig. 5b). The same dose mAb treatment on day 3 post-infection shown 60% protective efficiency in mice. Though therapeutic treatment did not prevent morbidity, as illustrated by the initial weight loss, all mice in these treatment groups regained and even exceeded their original body weight by the end of the 14 days observation period.

## Discussion

Similarly, H9N2 can cause human infections and may pose a severe public health threat, and attention needs to be paid for the pandemic preparedness [45]. Researchers have demonstrated that the widespread dissemination of H9N2 viruses poses a threat to human health not only because of the potential of these viruses to cause an influenza pandemic, but also because they can function as “vehicles” to deliver different subtypes of influenza viruses from avian species to humans [46]. The HA protein of influenza A virus serves as the primary target for neutralizing antibody production [44, 47], thus it is the most important antigen in IAVs. Traditionally, influenza vaccines mainly elicit strain-specific neutralizing antibodies against HA protein but generally show inefficient protection against newer strains due to antigenic drift or shift [20, 48]. To overcome the deficiency of these influenza vaccines, the development of broadly protective vaccines against multiple or all subtypes of IAVs is a desirable strategy. It is well established that the stem region of the HA protein is relatively conserved among all subtypes of IAVs. Properly leveraging this knowledge could lead to the development of a single vaccine that protects against most or all subtypes of IAVs. The first heterosubtypic monoclonal antibody isolated, C179, was elicited by hyperimmunization of mice with an H2N2-expressing human virus around 20 years ago [49]. Since then, a great many vaccine candidates with heterosubtypic activity have been generated, as described in the introduction section.

The broadly neutralizing antibodies generated against IAVs were comprehensively reviewed by Corti, et al. [50]. And the structural design of small proteins and peptides against the HA was comprehensively reviewed by Wu and Wilson [51]. Here, we produced a subtype cross-reactive monoclonal antibody 3C12, which was generated from an immortalized hybridoma cell line isolated following immunization of mice with bacterially-produced HA2 protein of AH/BRI99/16 (H9N2) virus. To identify the characteristics of this mAb, a series of experiments were performed in this study. We demonstrated that a linear neutralizing epitope ^95^NAELLVL^101^ (HA2 numbering) in the C-helix region of HA protein could be efficiently recognized by mAb 3C12. Compared with those antibodies have been produced in other literatures, this is the first linear cross-reactive epitope located in the C-helix region of HA2.

The above results indicated that mAb 3C12 could react with a majority of subtypes of group 1 including H1, H2, H5, H8, H9, H12, H13, H16 viruses and also the H18 HA protein. Interestingly, the H17 HA protein could react with 3C12 in WB but not in IFA, suggesting the targeting epitope of H17 protein might have a different three-dimensional conformation compare to other HA proteins in group 1. In addition to this, 3C12 also couldn’t react with the tested H6 strain, A/turkey/Massachusetts/3740/1965 (Massachusetts/3740/65, H6N2), in WB and IFA experiments, even though it shared the exact same linear sequence of HA2 from 95 to 101 (HA2 numbering) with other subtypes in group 1. We speculated that there might be other amino acids around this epitope spatially block the reactivity, but the specific residues and the underlying mechanism need further exploration. It is disappointing that the mAb does not react with subtypes of group 2. Modifications on this epitope may provide new ideas for the design of monoclonal antibodies to attain the cross-group reactivity as an influenza vaccine candidate in the future. To our knowledge, the identified linear neutralizing epitope located in the C-helix region of HA2 was partly in the internal of HA trimer. Indeed, a majority of cross-reactive HA mAbs, such as CR6261 [24, 25] and CR9114 [35], mainly recognize the fusion peptide and helix A region from the stem of HA. Therefore, our research might provide a novel region for generating broad-spectrum influenza vaccine candidates. Moreover, we found that in vitro 3C12 could inhibit the replication of most influenza subtypes of group 1, as measured by micro-neutralization assay in vitro. This mAb also had prophylactic and therapeutic protective activities in vivo. However, whether 3C12 has similar protective efficacy against other subtypes in group 1 needs further investigation.

Similarly, the FluA-20 was a broadly protective and naturally occurring human Ab. Researchers have demonstrated that the epitope which FluA-20 recognized was located in the internal of HA trimer, but it can reacted with nearly all subtypes of IAVs except for H16. The mechanism was that FluA-20 bond to the HA trimer interface and blocking cell-to-cell spread [52]. Another study reported the identification of three non-neutralizing but protective human Abs to H1 and H3 that bound to monomeric but not trimeric forms of HA [53]. In this study, the mechanism under the broad-spectrum activity of 3C12 had not been conducted. However, the two studies may provide some useful information to our following research.

## Conclusions

In this study, we confirmed that the broadly reactive monoclonal antibody 3C12 suppressed the replication of most of subtypes on group 1 in vitro and tested H9N2 subtype in vivo. This indicates that the broadly neutralizing antibody 3C12 may contribute to the development of therapies against H9N2 strains, and could prove effective protection against emerging strains of pathogenic H9. Furthermore, it has the potential to become a broad-spectrum influenza vaccine candidate against other IAVs in group 1 as suggested by the results of in vitro test. The novel HA2 C-helix epitope recognized by mAb 3C12 may become a new target region for the design of broad-spectrum monoclonal antibodies in the future and the underlying mechanism needed to be investigated in the near future.

## Data Availability

All relevant information is provided in this current manuscript. If required, the data presented in this work can be shared by e-mail.

## Abbreviations

WHO: World Health Organization
IAVs: Influenza A viruses
HA: Hemagglutinin
NA: Neuraminidase
MDCK: Madin-Darby canine kidney cells
ATCC: American Type Culture Collection
mAbs: monoclonal antibodies
MOI: Multiplicity of infection
IC_50_: 50% Inhibitory concentration
TCID_50_: 50% tissue culture infectious dose
iTOL: Interaction tree of life
